# Seasonal and Spatial Variability of Anthropogenic and Natural Factors Influencing Groundwater Quality Based on Source Apportionment

**DOI:** 10.3390/ijerph15020279

**Published:** 2018-02-06

**Authors:** Xueru Guo, Rui Zuo, Li Meng, Jinsheng Wang, Yanguo Teng, Xin Liu, Minhua Chen

**Affiliations:** 1 College of Water Sciences, Beijing Normal University, Beijing 100875, China; guoxueru@mail.bnu.edu.cn (X.G.); meng100li@sina.com (L.M.); wangjs@bnu.edu.cn (J.W.); teng1974@163.com (Y.T.); liuxin.good.good@163.com (X.L.); cmhsyg@163.com (M.C.); 2 Engineering Research Center of Groundwater Pollution Control and Remediation, Ministry of Education, Beijing 100875, China

**Keywords:** groundwater quality, groundwater-river water interaction, hydrochemistry, seasonal and spatial distribution, principal component analysis, factor analysis

## Abstract

Globally, groundwater resources are being deteriorated by rapid social development. Thus, there is an urgent need to assess the combined impacts of natural and enhanced anthropogenic sources on groundwater chemistry. The aim of this study was to identify seasonal characteristics and spatial variations in anthropogenic and natural effects, to improve the understanding of major hydrogeochemical processes based on source apportionment. 34 groundwater points located in a riverside groundwater resource area in northeast China were sampled during the wet and dry seasons in 2015. Using principal component analysis and factor analysis, 4 principal components (PCs) were extracted from 16 groundwater parameters. Three of the PCs were water-rock interaction (PC_1_), geogenic Fe and Mn (PC_2_), and agricultural pollution (PC_3_). A remarkable difference (PC_4_) was organic pollution originating from negative anthropogenic effects during the wet season, and geogenic F enrichment during the dry season. Groundwater exploitation resulted in dramatic depression cone with higher hydraulic gradient around the water source area. It not only intensified dissolution of calcite, dolomite, gypsum, Fe, Mn and fluorine minerals, but also induced more surface water recharge for the water source area. The spatial distribution of the PCs also suggested the center of the study area was extremely vulnerable to contamination by Fe, Mn, COD, and F^−^.

## 1. Introduction

Groundwater is indispensable for human survival and sustaining societal development. It is a ubiquitous source of water for domestic, agricultural, and industrial purposes. Nevertheless, an increasing trend of groundwater overexploitation and deterioration at both the regional and global scales, mainly due to anthropogenic activities, has recently been demonstrated [1,2,3]. Moreover, during the past few decades, climate change has gradually exacerbated the pressure on the hydrologic system and induced potential risks for drinking water safety, particularly in semi-arid and arid regions with considerable seasonal groundwater recharge and discharge [4,5]. Thus, there is an urgent need to quantify potential pollution sources and evaluate hydrogeochemical processes affecting groundwater quality for its sustainable application [6].

Natural climate change and seasonal variation dynamically impact groundwater recharge, runoff, and discharge conditions. In particular, seasonally different intensities in precipitation considerably influence both water quantity and quality [7]. In addition to natural influences, fluctuating water tables modified by anthropogenic activities in response to temporal and spatial variations can potentially degrade groundwater quality through more complex hydrogeochemical reactions [2]. Groundwater exploitation around a water source area leading to groundwater quality denigration and pollution risks, has also been documented in studies at the regional scale [8,9]. A declining water table due to exploitation will induce anaerobic conditions and higher hydraulic gradients in aquifer passages, which benefits water-rock interaction of soluble iron and manganese, fluorine and other metals into the groundwater [10]. Furthermore, the groundwater from a riverside water source area responds sensitively to seasonal precipitation owing to the close interaction between the groundwater and surface water. This results in changes to water levels, redox conditions, and diverse acidic and alkaline environments responsible for a myriad of geochemical reactions in a riverbank filtration system [11]. Apart from temporal variations, spatial land use types and management play an increasingly vital role in groundwater quality. In agricultural areas, seasonal nitrogen input from fertilizers and nitrogen transport in aquifers have been major threats to groundwater quality [12,13,14]. Therefore, evaluating seasonal and spatial variability of groundwater quality has gradually become the focus of research on groundwater source areas.

The chemical components of groundwater reflect the chemical compositions of the geologic formation lithology and the presence of contaminants from anthropogenic sources [15]. Natural sources include the geologic setting, water-rock interaction, marine water intrusion, bedrock weathering, and soil loss [16,17]. Anthropogenic sources include chemical industrial sources, mechanical processing, landfill leakage, fecal pollution, livestock wastewater, and agricultural pollution such as fertilizer application and irrigation water [6,18,19,20]. All the above-mentioned factors are usually identified using hydrochemical analyses and multivariate statistical methods. Depending on a large range of data, descriptive statistics, a piper trilinear diagram, ratios of major ions, correlation analyses, and isotopic trace analysis are common ways to determine groundwater origin and sources of its components [21,22]. Multivariate statistical methods, such as factor analysis (FA), cluster analysis (CA), principal component analysis (PCA), positive matrix factorization (PMF) and chemical mass balance (CMB) [18,23,24,25], have often been used as effective tools in the classification of samples and identification of pollution sources, by decreasing the dimensionality of many variables into a smaller set of common factors with minimum loss of information [26,27,28]. These dimension-reduction techniques can obtain the number of sources without a priori knowledge regarding their compositions [29]. This property is beneficial in evaluating water quality effectively and validating potential pollution sources.

The study area is adjacent to the Hulan River in Northeast China, and mainly receives concentrated rainfall during the wet season. The Hulan River has been severely polluted by large amount of waste water from upstream factories, including various contaminants of NO_3_^−^, NO_2_^−^, NH_4_^−^, suspended solids, turbidity, Fe, Mn, F^−^ and COD. Its water quality is poorer than the class V standard value of the Environmental Quality Standards for Surface Water (GB3838-2002) in China, which is not appropriate to be drinking water sources. In addition, the river flow cutoff is also a serious problem for stable water supply, which can last up to two months sometimes due to the drought weather. Therefore, the river water is only used for industrial purposes and agricultural irrigation in the study area, and the demand of domestic water supply for groundwater has been increasing remarkably [30]. The Hulan water source area, as an urban drinking water supply well field, was designed and built approximately along 3–4 km of the Hulan River with good water yield property and permeability in the study area. However, population pressure and industrial and agricultural development have resulted in more intense pressure on its exploitation than the groundwater recharge, especially in the dry season. A depression cone near the water source area is dramatic, which also causes groundwater deterioration. Therefore it is urgent and significant to understand the geochemistry of local groundwater from the seasonal and spatial perspectives by source apportionment for water supply sustainability and drinking water safety. The present study aimed to identify the seasonal and spatial variability of groundwater quality based on source apportionment in the Hulan water source area. The specific objectives were to: (1) identify the source apportionment of groundwater quality during the wet and dry seasons; (2) analyze the controlling hydrogeochemical processes and their spatial variation; and (3) compare the results of groundwater source apportionment during two seasons and provide recommendations for future environmental management.

## 2. Study Area

**Figure 1:**
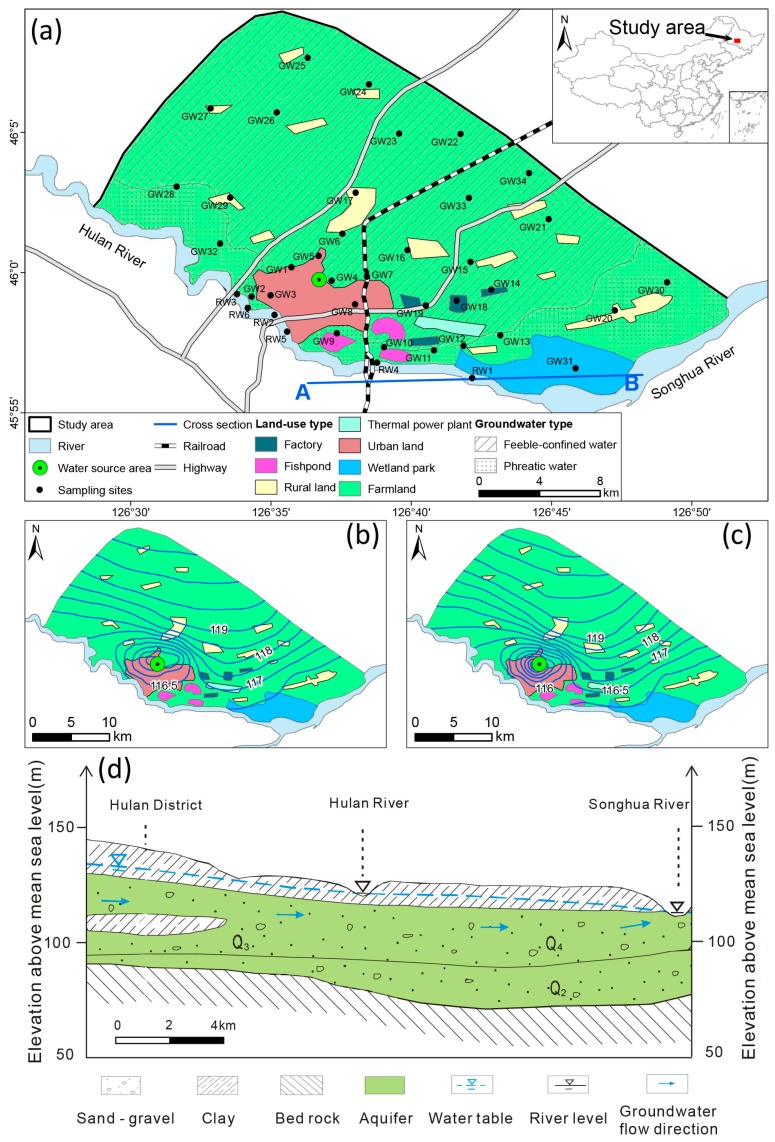
(**a**) Location of study area comprising the sampling sites, a typical cross-section of regional hydrogeology and land use types. Groundwater flow distribution during the (**b**) wet and (**c**) dry seasons, respectively; (**d**) Typical cross-section (A–B) of regional hydrogeology with the location plotted in Figure 1a.

**Figure 2:**
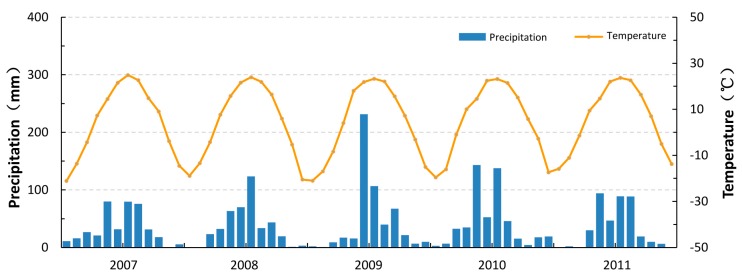
Cumulative rainfall and air temperature for the study area.

The study area is located in Hulan District of Harbin City, Heilongjiang Province, Northeast China. It is at the confluence of the Hulan and the Songhua Rivers (Figure 1). The Hulan River is an important tributary of the Songhua River. With a typical north temperate monsoonal climate, the study area has considerable seasonal variations in precipitation and temperature. In Figure 2, Distributions of local rainfall and air temperature are inhomogenous on time [30]. Most rainfall with higher temperature occurs from May to September (>50 mm). The highest temperature always appears in July (>23 °C) and the lowest one is in January (<−17 °C). Precipitation of <25 mm occurs from October to April. Rivers freeze in months with lower temperature <0 °C. Therefore, local seasonal variation is characterized by two periods, a wet season (May to September) and a dry season (October to April). The Hulan District beside the Hulan River has developed as a large urban region. Semi-urbanized and rural-industrialized locations mainly refer to the urban-rural adjacent areas, which include numerous factories including food processing, aquaculture farms, poultry farms and thermal power stations. Additionally, farm land and rural residential land is widely distributed in the study area. The Hulan River has been severely polluted by large amount of waste water from upstream factories, including various contaminants of NO_3_^−^, NO_2_^−^, NH_4_^−^, suspended solids, turbidity, Fe, Mn, F^−^ and COD. Its water quality is poorer than the class V standard value of the Environmental Quality Standards for Surface Water (GB3838-2002) in China, which is not appropriate to be drinking water sources. In addition, the river flow cutoff is also a serious problem for stable water supply, which can last up to two months sometimes due to the drought weather. Therefore, the river water is only used for industrial purposes and agricultural irrigation in the study area and the demand of domestic water supply for groundwater has been increasing remarkably.

The Hulan water source area, as an urban water supply well field, was designed and built approximately along 3–4 km of the Hulan River, with total yield of 3.3 × 10^4^ m^3^/day. According to groundwater level surveys during the two seasons, it was found that the groundwater flows from the north (high plain region) to south (the Hulan River) and southeast (the Songhua River) at a shallow groundwater depth of less than 10 m in general (Figure 1b,c). The surveys also qualitatively estimated the varying flowpath and facilitated a better definition of the extent of the regional hydrological system [6]. Groundwater levels and flow directions in the area have been drastically modified by extraction of groundwater (Figure 1b,c), which has stimulated more lateral flow from groundwater and river water, and development of a clear depression cone.

Regional shallow groundwater is mainly stored in a Quaternary formation with a thickness of approximately 15–30 m (Figure 1a), and is the primary source of water for domestic use in the study area. Three types of deposits from the middle Pleistocene (Q_2_), late Pleistocene (Q_3_), and Holocene (Q_4_) are shown in Figure 1d. They are primarily composed of gravels and sands with high porosity and good permeability (Figure 1d). According to previous data, the sedimentary rocks are composed of minerals such as calcite, dolomite, gypsum, feldspar, and fluorine and ferromanganese nodules [31,32]. Weathering and dissolution of these minerals influence the evolution of the groundwater chemistry. Bicarbonate water is the major water type of the local water. In addition to natural factors, local water is also polluted by anthropogenic activities including domestic sewage and agricultural wastewater [30].

## 3. Materials and Methods

### 3.1. Sample Collection and Analysis

A total of 34 shallow groundwater samples were collected at a determined depth from 2 to 6 m beneath the groundwater and six river water samples were taken at midstream level during the wet (August) and dry (November) seasons in 2015. At the same time, sediment samples were taken from the sand-gravel aquifer, distributed along the Songhua and Hulan Rivers. Locations and elevations of the sampling points were recorded by high precision GPS (SOUTH YINHE6), and the groundwater depths were measured by Water Level Meter (SOLINST (101) Georgetown, Ontario, Canada). For ensuring representative groundwater samples, the wells were pumped continuously to clean water before sampling, and samples were filtrated through a 0.45 μm filter membrane and filled in a 125 mL bottles. All bottles were completely filled with water without headspace sealed with a parafilm tape and stored in a refrigerator at 4 °C until analysis. Temperature and pH were in-situ measured using a EUTECH PCD 650 portable multi-parameter instrument (Thermo Fisher Scientific, Shanghai, China). All water samples were analyzed at the Beijing Center for Physical & Chemical Analysis in China. Indexes of potassium (K^+^), sodium (Na^+^), calcium (Ca^2+^), magnesium (Mg^2+^), chloride (Cl^−^), sulfates (SO_4_^2−^), nitrates (NO_3_^−^), nitrites (NO_2_^−^), ammonia nitrogen (NH_4_-N) and fluorine (F^−^) were analyzed by Ion Chromatography (ICS1100, Thermo Fisher Scientific, Shanghai, China). Bicarbonate (HCO_3_^−^) was analyzed by acid base titration. Based on charge–balance calculation, test error was controlled to <5%. Iron (Fe) and manganese (Mn) was analyzed by ICP-MS (7900, Agilent, Beijing, China). Chemical oxygen demand (COD), total dissolved solid (TDS) and total hardness (TH) were analyzed according to the Chinese Quality Standard for Groundwater (GB/T 14848-93). Sediment samples were analyzed at the Analytical and Testing Center of Beijing Normal University in China.

Samples of groundwater and river water in the wet and dry seasons for environmental isotope analyses (^18^O and ^2^H) were also taken in the field. To prevent potential isotope fractionation during storage, sample bottles were completely filled with water without headspace and sealed with a parafilm tape. All samples were stored in bottles at 4 °C until laboratory analyses. The river water and groundwater samples were analyzed in the Analytical Laboratory of the Beijing Research Institute of Uranium Geology in China with continuous flow analysis test of ^2^H and O isotope in water [33]. The results of stable isotopic analysis are expressed conventionally as *δ* values, representing deviation per mil (‰) from the isotopic composition of a specified standard (Vienna standard mean ocean water):

*δ*^18^O(*δ*^2^H) = [(*R*_sample_/*R*_standard_) − 1] × 1000‰ (1)

where *R* refers to the ^2^H/^1^H(^18^O/^16^O) ratio in a sample and standard. The measurement was consistently ±1‰ for *δ*^2^H and ±0.3‰ for *δ*^18^O. The correlation between *δ*^18^O and *δ*^2^H in precipitation on a global scale is well known as GMWL [34]. The stable isotope composition of rainfall in a region may represent different meteoric conditions and is better described by LMWL, which may have a slightly different slope and intercept compared with the GMWL [35].

According to the hydrochemical properties and the hydrogeological condition, 16 groundwater quality indexes were selected including K^+^, Na^+^, Ca^2+^, Mg^2+^, HCO_3_^−^, Cl^−^, SO_4_^2−^, NO_3_^−^, NO_2_^−^, NH_4_-N, Fe, Mn, F^−^, COD, TDS and TH. Based on the continuity of the sampling results, PCA/FA analysis was used to distinguish the impacts of different types of pollution (natural and anthropogenic) to groundwater quality in the Hulan groundwater source area. The employed pretreatment method and its suitability to the data are vital for application of PCA to the environmental datasets. In this study, pretreatment methods for the data, such as the elimination of non-informative variables, the treatment of missing data values, and the detection and treatment of outliers were performed prior to the PCA/FA analysis.

### 3.2. Principal Component Analysis and Factor Analysis

The principal component analysis (PCA) and factor analysis (FA) methods are mathematical tools used to reduce the description of variable dimensions for the easy understanding and analysis of the dataset. In reality, a PCA can be regarded as a special FA case. The PCA is a multivariate statistical analysis method that depends on an orthogonal transformation to convert a set of observations, for possibly correlated variables, called principal components (PCs) [36]. The principal component could be expressed as:

(2) $$\left\{\begin{array}{c} X_{1}=\partial_{11}F_{1}+\partial_{12}F_{2}+\ldots +\partial_{1m}F_{m}\\ X_{2}=\partial_{21}F_{1}+\partial_{22}F_{2}+\ldots +\partial_{2m}F_{m}\\ \ldots \\ X_{n}=\partial_{n1}F_{1}+\partial_{n1}F_{2}+\ldots +\partial_{nm}F_{m} \end{array}\right\}$$

(3) $$X=AF+E,A={\left(\partial_{ij}\right)}_{n\times m}$$

where, *X*_n_ is the component score, ∂ is the component loading, *F* is the measured value of variable, *n* is the component number, *m* is the total number of variables. In PCA, the principal component of eigenvalue is greater than unity, which is generally considered as the highest variability from the original dataset.

FA is similar to principal component analysis on the principle of calculation. It is an internal analysis that uses a lower number of unobserved variables, called factors, to explain the more complex relationships in observed variables [37]. The original loadings are usually rotated, implying that each variable has a very high factor loading (as high as 1) on one of the PCs, and a very low factor loading (as low as 0) on the other PCs. In this study, the PCA/FA was performed on normalized variables. The FA can therefore be expressed as:

(4) $$Z_{ij}=\partial_{f1}f_{1i}+\partial_{f2}f_{12}+\partial_{f3}f_{13}+\ldots +\partial_{fm}f_{1m}+e_{\mathrm{fi}}$$

where, ∂ is the component loading, *f* is the factor score, *e* is a residual term which accounts for errors or another source of variation, *i* is the sample number and *m* is the total number of variables.

An important precondition of the PCA and FA, is for the original variables to have a strong correlation. The Kaiser-Meyer-Olkin (KMO) and Bartlett’s tests were used to evaluate the relationship between the correlation coefficients and partial correlation coefficients of the original variables. Generally, the KMO statistic varies between 0 and 1. Kaiser recommended accepting values greater than 0.5. Furthermore, values between 0.5 and 0.7 were deemed “mediocre,” values between 0.7 and 0.8 were “good,” values between 0.8 and 0.9 were “great,” and values above 0.9 were “superb”. Bartlett’s test of sphericity was used to test for variable dependency. If the statistical result of the Bartlett’s test was large, the dataset was considered suitable for the factor analysis. Conversely, if the correlation coefficient matrix was not a unit matrix, the dataset was not considered suitable for a factor analysis.

## 4. Results and Discussion

### 4.1. Water Chemistry

A summary of water quality parameters of groundwater and river water during the wet and dry seasons (August 2015 and November 2015) of the study area is shown in Table 1. The temperature of groundwater was generally 7–10 °C and the pH was between 6.8 and 8.0. Average concentrations of major ions in the groundwater during the two seasons were generally similar, because mean values were close to the median (Table 1). The major cation and anion were Ca^2+^ and HCO_3_^−^, respectively, indicating the major minerals of carbonates. Standard deviations of Ca^2+^ and HCO_3_^−^ changed substantially in the two seasons, which may result from the spatial variability of the sediments and lithology, and some influence of human activities at the same time. A comparison of groundwater samples during the wet and dry seasons indicated higher concentrations of constituents during the dry season, including Ca^2+^, Na^+^, Mg^2+^, HCO_3_^−^, SO_4_^2−^, Cl^−^, and TDS. With regard to the average concentrations of total nitrogen (TN), NO_3_^−^ was the most remarkable, followed by NH_4_^+^ and NO_2_^−^. Meanwhile, NO_3_^−^ during both seasons far exceeded the limitation value (20 mg/L) for drinking water quality [38], suggesting severe nitrogen pollution of the local groundwater. The higher standard deviations of 108.58 and 75.09 of NO_3_^−^ during the wet and dry seasons, respectively, indicated that there was a large deviation between the samples possibly due to seasonal anthropogenic activities. The mean value of Fe and Mn are 5–7 mg/L, 0.8–1.0 mg/L, respectively, suggesting the serious contamination of Fe and Mn in local groundwater. The mean value of COD was also strikingly different, owing to the standard deviations of 2.94 and 0.46 during the wet and dry seasons, respectively. The mean value of F^−^ (0.73) during the dry season was two times than that during the wet season. This water pollution situation in the study area suggested that domestic sewage and agricultural wastewater had a large effect on local groundwater quality [30].

The river water was weakly alkaline, with pH~7, and its temperature changed considerably, from approximately 26–28 °C during the wet season to 2–8 °C during the dry season. The ion concentration in the river water during the two seasons was nearly the same on average, except for Mg^2+^ during the dry season. Characteristic pollutants included NO_3_^−^, NH_4_^+^, and NO_2_^−^ and the COD concentrations proved the river was seriously polluted. Comparing the values of river water, the mean concentration of NO_3_^−^ (14.49 mg/L) during the dry season was 3 times more than the 4.02 mg/L recorded during the wet season. From the mean value of Fe and Mn (1–2 mg/L, 0.1–0.2 mg/L, respectively), they are also the contaminants in river water. However, during the wet season, the COD was usually two times that during the dry season, showing a positive relation with higher temperature during the wet season.

### 4.2. Source Apportionment of Groundwater Pollution

#### 4.2.1. PCs during the Wet Season (PC-W) and Dry Season (PC-D)

In the groundwater environment disturbed by anthropogenic activities, PCA combined with the FA technique can highlight those chemical parameters of groundwater from the pervasive natural background. In this study, Kaiser–Meyer–Olkin (KMO) and Bartlett’s sphericity tests were applied to examine the suitability of the data during the wet and dry seasons by PCA/FA. Ion contents of local shallow groundwater during the wet and dry seasons were analyzed with results of 0.511 and 0.547 (>0.5) respectively (Table 2). The results revealed a close relationship between ion types and these were suitably selected as variables for multivariate statistical analysis.

PCA/FA was used to identify the importance of parameters by separating classes. The chemical compositions controlling the chemical variability were extracted from the groundwater samples, and the rotation of PCs was carried out using the varimax method. According to the results of PCA/FA, only four of the variables displayed an eigenvalue higher than 1.0, and the eigenvalue of the remaining variables changed insignificantly. The four selected factors were sufficient for the investigation of pollution sources. During the wet season, PC_1_-W, PC_2_-W, PC_3_-W, and PC_4_-W explain 77.45% of the total variance (Table 3).

PC_1_-W, explaining 37.97% of the cumulative variances was mainly driven by Ca^2+^, Mg^2+^, Na^+^, Cl^−^, SO_4_^2−^, and TH with loadings between 0.702 and 0.90. The metal elements Fe and Mn contributed most strongly to the second component (PC_2_-W), explaining 20.85% of the total variance. PC_3_-W with the variables NO_3_^−^, K^+^, and NH_4_-N (with loadings between 0.777 and 0.969), represented 10.70% of the total variance in the original data set. Finally, the COD contributed most strongly to PC_4_-W explaining 7.93% of the total variance (Table 3), which approximately indicated the total amount of organic matter in the groundwater. During the dry season, four PCs were extracted with a cumulative variance of 71.69% (Table 3). PC_1_-D was the first factor influencing the groundwater quality of the study area, and accounted for 27.53% of the total variance with an eigenvalue of 6.06. This was analogous to the first factor with similar sources of pollution during the wet season. PC_2_-D had strong and positive loadings on Mn and Fe accounting for 17.40% of the total variance. PC_3_-D representing 15.95% of the total parameters was mainly related to components NH_4_-N, NO_3_^−^, and NO_2_^−^. PC_4_-D explained 11.82% of the total variance with F^−^ (Table 3).

#### 4.2.2. Source Apportionment with Spatial Distribution of PCs

**Figure 3:**
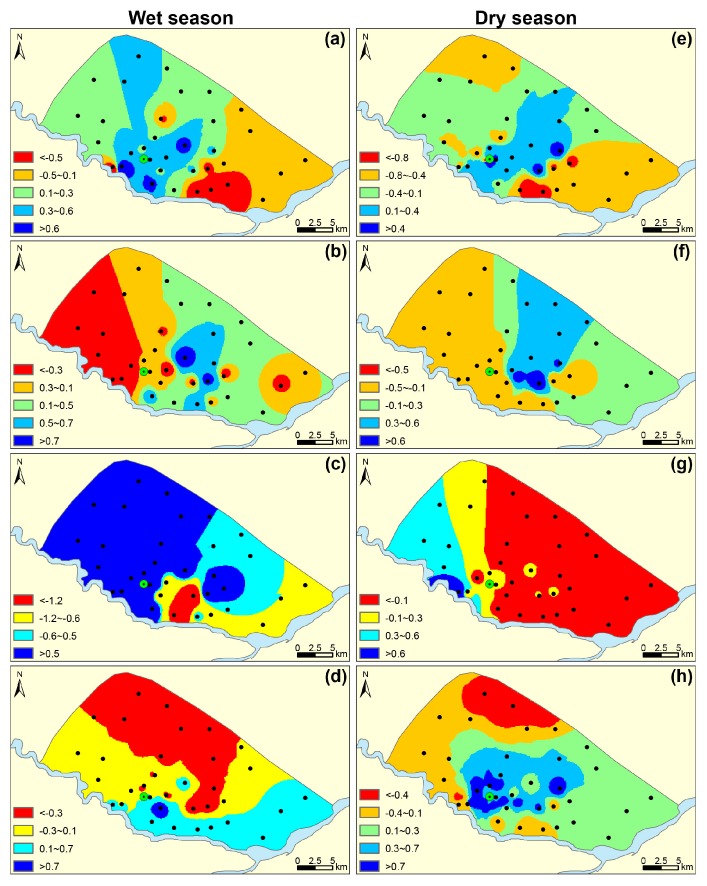
Spatial distribution of PCs. Wet season: (**a**)-PC_1_-W; (**b**)-PC_2_-W; (**c**)-PC_3_-W; (**d)**-PC_4_-W; Dry season: (**e**)-PC_1_-D; (**f**)-PC_2_-D, (**g**)-PC_3_-D; (**h**)-PC_4_-D.

In order to obtain a sound understanding of the original variables and significances of the PCs, varimax component scores of groundwater samples were separately interpolated for each PC (PC_1_, PC_2_, PC_3_, and PC_4_) on a regular grid using ArcGIS 9.3 software (2009, ESRI, Redlands, CA, USA). A higher factor score is associated with a more severe pollution grade. Extreme negative scores (<−1.0) reflect areas essentially unaffected by the process and positive scores (>+1.0) reflect areas most affected. Areas with near-zero scores mean it is influenced to an average degree by the chemical process of that particular PC. Figure 3 shows the spatial distribution of the component scores for each PC during the wet and dry season respectively of the study area.

• **PC1: Water-Rock interaction**

**Figure 4:**
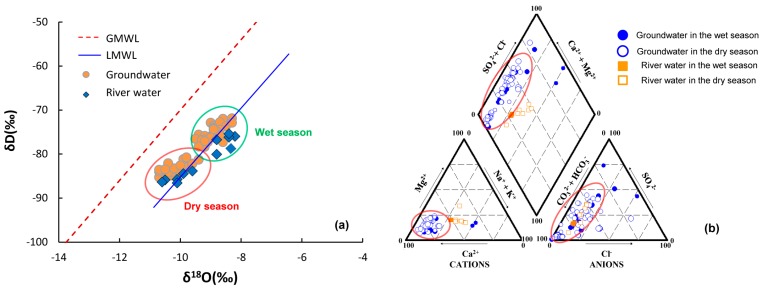
(**a**) Conventional diagram of *δ*^18^O/*δ*^2^H for groundwater and river water; (**b**) Piper trilinear diagram for water samples of the study area.

A spatial distribution of scores for PC_1_-W is presented in Figure 3a, where high scores were generally observed in the northern and central parts during the wet season, but the high factor score in the dry season had significantly expanded only in central part compared to the wet season. The graph of water samples distribution with the GMWL and the LMWL is the basis for stable isotope studies and to determine water origin. In Figure 4a, two groups of water samples were obvious, with the wet season on the upper right and the dry season on the lower left. During the wet season, *δ*^2^H and *δ*^18^O of groundwater varied from −77.3‰ to −72.1‰ and −9.4‰ to −8.3‰, respectively (Table 4). The sample data in the group were close to the local meteoric water line (LMWL) and relatively enriched, suggesting that the groundwater was derived from the infiltration of evaporated rainfall and most rainfall during the wet season was a conspicuous recharge for the local groundwater system. During the wet season, the water table elevation was approximately 114.7–120.2 m in the northern area and decreased toward the Hulan River, with mean hydraulic gradients of 0.03–0.04%. Therefore, due to the local general groundwater flowpath from north to south with high permeability of the aquifers (Figure 1), large precipitation recharge during the wet season was benefit for natural weathering processes and the dissolution equilibrium of reactive minerals (calcite, dolomite, and gypsum) into the groundwater [6]. A piper diagram is a useful tool to classify groundwater type and readily reveal its evolution by plotting the concentration of major cations and anions in graphical representations [21,22]. The lower left sample distribution of red circles in Figure 4b represented major bicarbonate water [39]. All of these proved that the classical chemical variables of Ca^2+^, Mg^2+^, SO_4_^2−^ and Cl^−^ in PC_1_ flowed with the groundwater flowpath by water-rock interaction [31].

**Figure 5:**
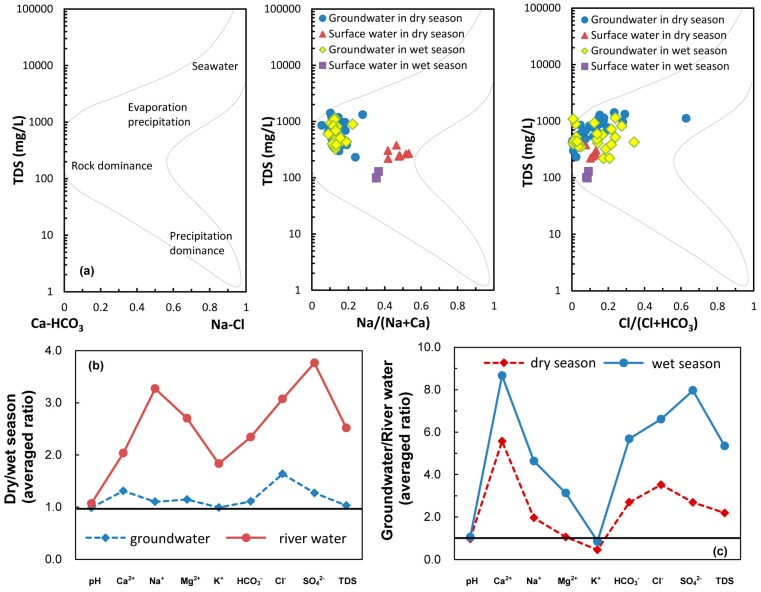
(**a**) Gibbs plots of groundwater and river water samples; (**b**) wet/dry average ratio of groundwater and river water; (**c**) groundwater/river water average ratio during the wet season and dry season.

The groundwater samples distributed in center of the Gibbs plots (Figure 5a) strongly favored water-rock interaction as the main process governing groundwater chemistry as an important source for K^+^, Na^+^, Ca^2+^, Mg^2+^, Cl^−^ and SO_4_^2−^ [40]. Samples outside of the red circles in Figure 4b with a higher concentration of SO_4_^2−^ and Cl^−^ in the groundwater and surface water, together with the spatial distribution of the highest score on the upper right near the factories (Figure 3a), both proved that the difference was caused by the influence of anthropogenic activities, namely industrial and domestic sewage discharging [30]. Owing to extraction in the groundwater source area, the depression cone apparently extended in the dry season. The hydraulic gradients of the depression cone in the dry season were much greater than that in the wet season. Intense evaporation and scarce precipitation decreased groundwater recharge and lateral flow in the dry season relative to that of the wet season. Additionally, seepage of irrigation water in the wet season resulted in groundwater recharge and weaker hydraulic gradient than in the dry season.

In Figure 4a of the dry season, *δ*^2^H of groundwater was between −84.7‰ and −80.8‰ and *δ*^18^O was from −10.6‰ to −9.4‰ (Table 4). The samples were relatively depleted, and together with those of river water were below the LMWL. This implies isotopic exchange in the aquifer and better hydraulic connection between the groundwater and river water during the dry season. As seen in Figure 5b,c, concentrations of major ions in river water and groundwater had an apparent response relationship. Ion concentrations of groundwater were larger than those in river water, and they were higher during the dry season, especially for river water. We explained this result as follows: for PC_1_-D, the lateral recharge of river water by groundwater extraction became one of the important sources. At the beginning of the infiltration, river water with lower concentrations (containing more CO_2_ and O_2_ and thus showing higher solubility) enhanced the water-rock interaction, and the highest score was observed at locations along the riverbank during the dry season (Figure 3e) [41]. On the other hand, the extraction promoted more lateral groundwater with a much higher hydraulic gradient of 0.16% during the dry season than that recorded during in the wet season (0.09%; Figure 1b,c). This phenomenon significantly accelerated the water-rock interaction with the highest score occurring near the water supply field (Figure 3e). The intensive dissolution of calcite, dolomite, gypsum, and potassium feldspar during the dry season resulted in different parameters of K^+^ and TDS for PC_1_-D. According to our investigation, the other two locations with the highest score were rural land and impacted by local factories. In such cases, industrial effluent and domestic sewage infiltration resulted in increasing components such as Ca^2+^, Mg^2+^, SO_4_^2−^, and Cl^−^ migrating to the groundwater easily (Figure 1). Therefore, PC_1_ was interpreted as the water-rock interaction influenced by seasonal precipitation, hydrological conditions, and anthropogenic activities.

• **PC2: Geogenic Fe and Mn sources**

**Figure 6:**
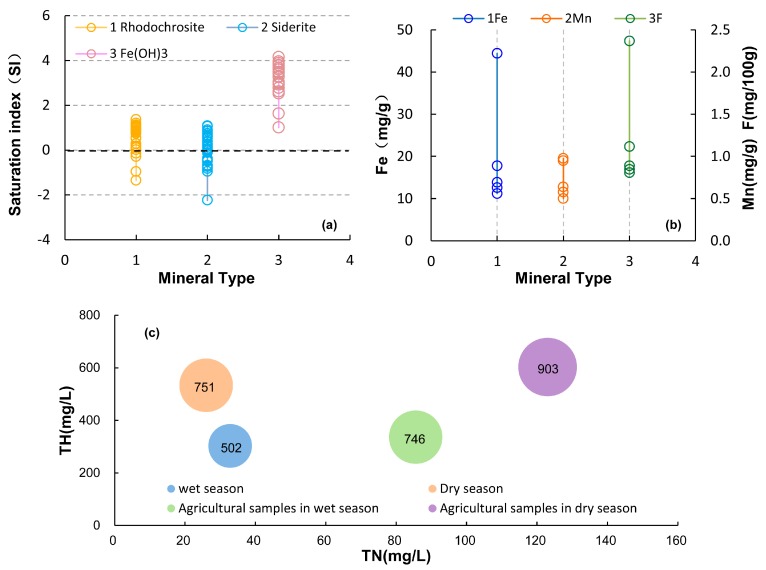
(**a**) Saturation indexes of minerals in groundwater samples; (**b**) Contents of Fe, Mn, and F in local sediments; (**c**) Bubble chart of total nitrogen and TH (the number at the bubble indicates the TDS concentration).

High contents of Fe and Mn in groundwater occurring in northeastern China have been reported in many studies, with high Fe concentrations generally occur where Mn concentrations are high [31,42,43]. The most concentrated area of Fe and Mn is in the center of the Songnen Plain, where our study area occurs [31]. The mean concentrations of Fe and Mn in local groundwater were 6.5 and 1.0 mg/L in the wet season (Table 1), far exceeding the guideline values of drinking water of 0.3 and 0.1 mg/L, respectively [38]. Local Quaternary aquifer sediments contained abundant iron–manganese nodules with contents of Fe and Mn as high as 11.2–44.4 mg/g and 0.5–1.0 mg/g, respectively (Figure 6b). The results of saturation indexes for local groundwater samples were calculated using PHREEQC shown in Figure 6a. Saturation indexes of rhodochrosite and siderite distributions fluctuated around the critical value of 0 but >0 in terms of Fe(OH)_3_, indicating that the groundwater was substantially in a dissolution equilibrium state with respect to rhodochrosite and siderite but highly oversaturated with respect to Fe(OH)_3_. The result was strong evidence for high contents of Fe and Mn in groundwater from dissolution of the original iron–manganese minerals [44]. The spatial distribution of higher scores was similar to the obvious groundwater flow direction, favoring the explanation for Fe and Mn dissolution of black soil as both variable valence elements (Figure 3b) [45]. The natural weathering and dissolution processes of the minerals are mainly responsible for the release of Fe and Mn; however, their activities are mostly controlled by the redox level of groundwater. The high concentrations of these elements were likely due to reductive dissolution of Fe/Mn oxides found in aquifer sediments [46]. This reducing environment was beneficial to the Fe and Mn contamination in upper layer of muddy loam and clay (Figure 1). The enrichment of organic matters was the result of fertilization in farm land and some factories in the central rural area, where a heterogeneous distribution occurred with the highest score [47]. In addition, the increase in NO_3_ concentrations in the pore water samples argued for a change in redox conditions during the wet season. This change might be related to the arrival of oxygen and the reacclimation of microbial populations [48,49]. On the other hand, the excessive exploitation of groundwater for irrigation induced a continuous decline in the water level during the wet. This also accelerated the reducing environment in the groundwater, providing good conditions for iron and manganese reduction reactions, and producing a larger blue-colored area with the highest score in the center. The specific reactions are as follows [50,51]:

Fe (OH)_3_ + 3H^+^ + e^−^ = Fe^2+^ + 3H_2_O; CH_2_O + Fe_2_O_3_ + 2H^+^ = 2Fe^2+^ + CO_2_ + 2H_2_O;

CH_2_O + 2MnO_2_ + 3H^+^ = 2Mn^2+^ + HCO_3_^−^ +2 H_2_O;

CH_2_O + 4Fe (OH) _3_ + 7H^+^ = 4Fe^2+^ + HCO_3_^−^ + 10H_2_O

During the dry season, mean concentrations of Fe and Mn in groundwater decreased to 5.2 and 0.9 mg/L respectively, compared to 6.5 and 1.0 during the wet season (Table 1). This trend can be explained not only by the weaker dissolution of the Fe and Mn minerals owing to less precipitation seasonally and slower groundwater runoff, but also by a change in the reductive conditions without fertilization and irrigation. The highest score during the dry season was downstream and its region was larger than that during the wet season, where it was in the rural area with many factories (Figure 3f). A large amount of industrial wastewater from these factories and domestic sewage in the rural area was an important source for the acceleration of the dissolution of the iron and manganese minerals [18]. Therefore, PC_2_ was primarily restricted to original geological Fe and Mn because of the unique hydrogeology and the reduction environment, and was mainly affected by agricultural activities during the wet season, and infiltration of anthropogenic sources and their spatial distribution during the dry season.

• **PC_3_: Agricultural contamination**

NO_3_^−^, K^+^ and NH_4_-N were contained in the PC_3_ in the wet season, which was similar with its components of NH_4_-N, NO_3_^−^ and NO_2_^−^ in the dry season (Table 3). According to the spatial distribution of PC_3_-W, the high score area was widely concentrated in western area, primarily agricultural land (Figure 1 and Figure 3c). It revealed that groundwater quality in the study area deteriorated in the wet season. However, the pollution area of dry season got shrunk greatly in the northwest of the research area, and the highest score located beside the Hulan River and near the groundwater source area.

The agricultural activity of fertilizer application is suspected to be the major nonpoint source contributing to nitrogen pollution of surface water and groundwater in northeastern China [14]. As seen in Figure 6c, the amount of TH and TDS increased with TN, and the values of TN were 33 and 26 mg/L during the wet and dry seasons, respectively. The highest concentration of TN in the agricultural area during the wet season was 123 mg/L and 86 mg/L for samples in the agricultural area during the dry season. These values were mainly controlled by the large amount of NO_3_^−^, which was expressed as the following chemical reactions:

$$\begin{array}{l} 2NH_{4}^{+}+3O_{2}\overset{\mathrm{nitriyr}\;\mathrm{bacteria}}{\to }2NO_{2}^{-}+2H_{2}O+4H^{+}\\ 2NO_{2}^{-}+2O_{2}\overset{\mathrm{nitrifying}\;\mathrm{bacteria}}{\to }2NO_{3}^{-} \end{array}$$

These increased variations in TN provided reliable indicators for contamination of groundwater by anthropogenic pollution, especially agricultural activities [39,52]. In nature, the presence of protein organic matter in groundwater usually results from fertilizer and manure application, whose decomposition is associated with nitrate, ammonia nitrogen, and nitrite formation. Therefore, a large amount fertilizer (K_2_SO_4_; NH_4_NO_3_; (NH_4_)_2_SO_4_) and manure application infiltrated into the groundwater by the intensive precipitation during the wet season, and finally increased the number of NO_3_^−^, K^+^, and NH_4_^+^ ions [14]. In the rural area, considerable domestic sewage and residential garbage containing large amounts of nitrogenous organic matter and microorganisms entered the groundwater either by rain filtration or was directly discharged into the ground or ditches without treatment [53].

During the dry season, mean values of TN were lower than those during the wet season, showing weaker nitrogen contamination from agricultural activities (Figure 6c). Together with the lower precipitation benefiting the infiltration of nitrogen, the higher score (>0.3) of the area distinctly decreased (Figure 3g). However, the concentrations of TN in the agricultural area during the dry season were much higher than those during the wet season (Figure 6c). The highest score was noted for agricultural land beside the Hulan River and near the groundwater source. The mean concentration of TN in river water was approximately 15.7 mg/L during the dry season, nearly four times higher than the 4.1 mg/L value during the wet season (Figure 6c). As previously mentioned, the highly polluted river water stimulated filtration into the groundwater system near the river, with high TN content owing to the exploitation of the water supply field and greater hydraulic gradients. To summarize, PC_3_ was interpreted as non-point source pollution of agricultural activities. Agricultural contamination was the most important source for high concentrations of nitrogen with considerable influence from excessive fertilizer applications during the wet season, and contaminated river water recharge near the Hulan River during the dry season.

• **PC4: Organic pollution (wet season) and Geogenic F enrichment (dry season)**

Finally, the PC_4_ represented the minimum percentage of the total variance but had different loadings on COD (7.93%) during the wet season and F^−^ (11.82%) during the dry season. The spatial distribution of COD showed that the areas with a high factor score (>0.1) were mainly located in southeast close to the Hulan River (Figure 1). But the areas with high factor score (>0.3) of F^−^ significantly distributed in the center during the dry season.

COD is an indicator of organic pollution from agricultural, industrial, and domestic wastewater. The urban area beside the river discharges large amounts of water pollutants causing deterioration of surface water quality [18]. Numerous small factories such as paper mills and metal-processing plants are near the rivers and directly discharge wastewater into the rivers without treatment in the study area [30]. As can be observed in Figure 3d, the river water has a much higher mean concentration (8.9 mg/L) of COD than the 2.6 mg/L of groundwater (Table 1). The high COD of the shallow groundwater in the downstream farmland beside the river can be attributed to irrigation by river water over a long period of time according to the investigation. Meanwhile, the majority of higher score area was originally old channels of the river with much riverbed sediment containing a considerable amount of organic soil in the stratigraphy; thus, the presence of relatively high COD concentrations was geologically derived as well. In addition, a wetland park had been built atop the old channels, which produced considerable volumes of organic wastewater from anthropogenic activity. This wastewater also caused groundwater contamination, particularly during the wet season [54]. Additionally, some food processing factories, aquaculture farms, poultry farms and thermal power stations (Figure 1), also produced a large amount of organic wastewater discharge in the area [30]. Furthermore, there were also domestic garbage stations containing organic matter with a large number of microorganisms, which have caused groundwater pollution to a certain degree. The highest score during the wet season was located at the fishponds and was largely related to excessive organic fish feed easily discharging into the groundwater. The concentration of COD varied closely with the seasonal temperature. The natural higher temperature was of benefit for its sources and reaction, and the major negative anthropogenic effects were concentrated during the wet season, thus contributing to the difference in PC_4_ between the two seasons [18,27,55,56]. Therefore, PC_4_-W during the wet season represented organic pollution in groundwater.

During the dry season, as mentioned previously, the intensive water-rock interaction around the water source with higher hydraulic gradients was the most significant difference relative to the weaker organic pollution observed under low-temperature conditions without irrigation during the wet season. Besides the minerals of the major ions (Fe and Mn) in the groundwater, it is well known that geological formations in northeastern China contain abundant fluorite and fluorapatite [43]. As can be observed in Figure 6b, there were high contents of fluorine from 0.8 to 2.41 mg/100 g and with a mean value of 1 mg/100 g in the local sediments. Fluorite minerals have been found in many regions of the study area [32]. In particular, the highest fluorine score distribution was apparently around the Hulan groundwater source area, where the higher hydraulic gradients enhanced the dissolution of fluoride minerals from the soil to the groundwater (Figure 3h). The similar radius of F^−^ and OH^−^, coupled to the same valence state, made the substitution possible in the minerals. The excessive OH^−^ of the local alkaline water replaced the F^−^ in the minerals and caused an increase in F^−^ concentrations in the groundwater source area [43]. Therefore, the high permeability of the groundwater system, the alkaline water environment (Figure 4b), and the dry weather conditions all benefited its dissolution (Section 2). Meanwhile, the other highest score encircled the factories because of wastewater sewage increasing the content of F^−^. Hence, the enrichment of fluorine in the groundwater was initiated in two favorable ways; the easy migration of fluorine in the alkaline water, and the rich content of fluorine in local minerals. Therefore, PC_4_-D was termed geogenic F enrichment mainly owing to the weathering and dissolution of fluorine minerals.

## 5. Conclusions

The results showed four anthropogenic and natural factors affecting the groundwater chemistry during the wet and dry seasons in the Hulan area. The water-rock interaction, dissolution from geogenic characteristic pollutants, and non-point source pollution of agricultural activities were major hydrochemical processes controlling the local groundwater quality during both seasons. The water-rock interaction was affected by seasonal precipitation, hydrological conditions, and the exploitation of groundwater. The geogenic Fe and Mn contamination were primarily influenced by agricultural activities during the wet season, and derived from infiltration of anthropogenic sources for their spatial distribution during the dry season. The agricultural contamination resulted from excessive fertilizer application during the wet season, and contaminated river water recharge near the Hulan River during the dry season.

The remarkable difference was organic pollution during the wet season and geogenic F enrichment during the dry season influencing the groundwater quality, which was a mutual superposition of the geogenic and anthropogenic factors. The naturally higher temperature and major negative anthropogenic effect concentrated during the wet season resulted in serious organic pollution. However, geogenic F enrichment was mainly derived from the weathering and dissolution of fluorine minerals resulting from the exploitation of groundwater during the dry season. From the spatial distribution of the PCs during both seasons, this study found that four PCs all have higher scores with major ions and contaminants (Fe, Mn, COD, and F^−^) in the central part of the study area. Diverse pollutants in wastewater infiltrated into the groundwater from various land types, including urban areas, rural areas, agricultural lands, and numerous factories, and thus, more attention should be paid to groundwater protection in these areas. Seasonally natural factors controlled the original groundwater flow and chemistry, and spatially anthropogenic activities intensified the hydro-geochemical reactions and produced various pollution sources deteriorating groundwater quality.

Some measures should be taken to control the groundwater pollution for water environmental protection and management due to the seasonal and spatial impact on local groundwater quality: (1) For industrial wastewater, improve wastewater treatment, reuse system to avoid various pollution sources into groundwater; (2) For domestic wastewater, build drainage system and centralized treatment plants to reduce the pollution from breeding industry in rural area; (3) For agricultural non-point pollutions especially in the wet season, regulate amounts and types of chemical fertilizers, and promote advanced ecological compensation and engineering technology for its appropriate application in the area; (4) In the central area, improve the removal efficiency of the Fe, Mn, F^−^ and nitrogen of the groundwater, and reinforce protections of environment encircling the groundwater source area to ensure the drinking water safety; (5) For river water pollution, strengthen the supervision of the discharge from upstream factories and prevent the discharge of wastewater which was not up to the effluent standards.

## Figures and Tables

**Table 1:** Comparison of groundwater and river water quality indexes during wet and dry seasons.

	Indexes	Min		Max		Mean		Med		S.D		CV	
	Seasons	WS	DS	WS	DS	WS	DS	WS	DS	WS	DS	WS	DS
GW	pH	7.06	6.91	7.97	7.94	7.68	7.58	7.78	7.60	0.31	0.29	0.04	0.04
	T	7.30	4.30	15.30	10.00	8.45	7.27	8.00	7.15	1.60	1.74	0.19	0.24
	Ca^2+^	18.60	46.80	265.00	298.00	137.79	180.33	153.00	170.00	2.47	72.62	0.02	0.40
	Mg^2+^	5.28	3.96	36.60	44.30	17.34	19.84	14.20	18.45	0.75	9.94	0.04	0.50
	Na^+^	15.70	12.80	54.70	96.50	27.94	30.76	17.50	26.60	0.84	19.73	0.03	0.64
	K^+^	0.56	1.22	6.50	3.47	2.04	2.02	1.54	1.95	0.25	0.64	0.12	0.31
	HCO_3_^−^	12.30	125	678.00	667.00	378.14	419.22	328.00	379.50	3.62	148.15	0.01	0.35
	Cl^−^	2.10	1.31	120.00	211.00	41.53	67.93	52.90	50.40	1.45	58.83	0.04	0.87
	SO_4_^2−^	3.75	9.55	318.00	289.00	95.59	121.40	138.00	122.50	2.35	75.16	0.02	0.62
	TDS	218.00	232.00	1140.00	969.00	585.00	602.50	597.00	604.00	4.89	215.77	0.01	0.36
	F^−^	0.15	0.16	0.41	0.73	0.30	0.34	0.32	0.33	0.07	0.11	0.24	0.31
	COD	0.56	0.29	11.40	2.92	2.55	1.81	1.60	1.83	2.94	0.46	1.16	0.25
	NO_3_^−^	2.14	1.19	394.00	230.00	32.40	25.34	10.33	7.60	108.58	75.09	1.90	1.67
	NO_2_^−^	0.00	0.00	1.09	0.23	0.20	0.07	0.02	0.04	0.34	0.08	1.73	1.04
	NH_4_^+^	0.05	0.03	5.67	1.95	0.40	0.59	0.10	0.58	1.12	0.38	2.81	0.64
	Fe	0.03	0.02	20.70	21.20	6.53	5.20	3.33	5.63	5.92	5.32	1.14	0.81
	Mn	0.03	0.02	3.32	3.66	1.00	0.89	0.95	0.77	0.79	0.70	0.79	0.78
RW	pH	7.15	7.62	7.50	8.04	7.31	7.80	7.29	7.82	0.18	0.14	0.02	0.02
	T	26.30	2.70	28.00	8.20	27.20	5.69	27.30	5.40	0.85	1.92	0.03	0.34
	Ca^2+^	13.90	27.10	19.50	44.70	15.90	32.36	14.30	30.30	3.12	6.65	0.20	0.21
	Mg^2+^	3.24	6.88	4.64	24.70	3.74	10.11	3.34	7.91	0.78	6.46	0.21	0.64
	Na^+^	7.60	19.50	11.30	38.80	8.93	29.20	7.88	27.50	2.06	6.57	0.23	0.23
	K^+^	2.25	4.00	2.54	5.18	2.38	4.36	2.35	4.24	0.15	0.40	0.06	0.09
	HCO_3_^−^	55.90	109.00	80.70	323.00	66.50	155.86	62.90	126.00	12.79	76.64	0.19	0.49
	Cl^−^	5.22	12.50	8.12	26.90	6.28	19.30	5.50	18.20	1.60	5.38	0.25	0.28
	SO_4_^2−^	11.10	22.90	13.80	68.10	12.00	45.19	11.10	39.80	1.56	16.39	0.13	0.36
	TDS	98.00	220.00	129.00	378.00	109.33	275.43	101.00	264.00	17.10	52.5	0.16	0.1
	F^−^	0.21	0.22	0.26	0.61	0.23	0.35	0.22	0.33	0.03	0.13	0.12	0.36
	COD	7.52	4.14	10.30	5.94	8.91	5.13	8.91	5.10	1.97	0.59	0.22	0.12
	NO_3_^−^	3.93	5.06	4.18	6.71	4.02	14.49	4.06	5.40	0.18	0.69	0.04	0.12
	NO_2_^−^	0.01	0.01	0.04	0.10	0.02	0.03	0.02	0.02	0.02	0.03	0.83	0.96
	NH_4_^+^	0.05	0.30	0.07	4.76	0.06	1.18	0.05	0.71	0.01	1.59	0.21	1.34
	Fe	0.01	0.69	2.41	3.06	1.39	1.98	1.74	1.87	1.24	0.80	0.89	0.41
	Mn	0.07	0.14	0.13	0.22	0.10	0.19	0.10	0.21	0.03	0.03	0.29	0.18

Units: T (°C), other indexes (mg/L) except for pH; GW: groundwater, RW: river water. Every first column: wet season (WS); Every second column: dry season (DS). Med: median; S.D: standard deviation; CV: coefficient variation.

**Table 2:** Results of the KMO and Bartlett tests during the wet and dry seasons.

Kaiser-Meyer-Olkin Measure of Sampling Adequacy		Wet Season	Dry Season
		0.511	0.547
Bartlett’s Test of Sphericity	The approximate chi-square	1571.422	2049.188
	Degrees of freedom	210	210
	Significance level	0	0

**Table 3:** Component loadings for the groundwater parameters during the wet and dry seasons.

Parameters	Components in the Wet Season				Components in the Dry Season			
	PC_1_-W	PC_2_-W	PC_3_-W	PC_4_-W	PC_1_-D	PC_2_-D	PC_3_-D	PC_4_-D
K^+^	0.323	0.003	**0.90**	−0.099	**0.63**	−0.14	0.89	0.34
Na^+^	**0.870**	0.093	0.149	−0.195	**0.81**	−0.08	−0.01	−0.08
Ca^2+^	**0.900**	0.261	0.088	0.070	**0.88**	−0.18	−0.25	0.05
Mg^2+^	**0.885**	0.119	0.219	−0.057	**0.93**	0.24	0.08	−0.11
NH_4_-N	0.043	0.004	**0.969**	0.042	0.39	−0.33	**0.83**	0.17
HCO_3_^−^	**0.820**	−0.341	0.411	−0.090	0.11	0.21	0.49	0.23
Cl^−^	**0.764**	0.441	−0.152	0.211	**0.85**	−0.03	0.31	−0.07
SO_4_^2−^	**0.801**	0.431	−0.127	0.202	**0.77**	0.24	0.38	0.01
NO_3_^−^	−0.158	0.261	**0.777**	−0.172	0.35	0.01	**0.84**	0.03
NO_2_^−^	0.131	0.104	0.150	−0.338	−0.22	0.30	**0.81**	0.19
Fe	0.220	**0.624**	0.258	0.2	−0.58	**0.65**	0.28	0.22
Mn	0.582	**0.578**	0.063	0.078	0.23	**0.78**	−0.81	0.11
F^−^	0.471	−0.795	−0.085	0.072	0.10	0.32	0.43	**0.84**
TH	**0.702**	0.021	−0.035	0.048	**0.93**	−0.11	−0.18	−0.03
COD	0.034	0.611	0.027	**0.921**	−0.29	0.45	0.44	−0.11
TDS	0.323	0.003	0.912	−0.099	**0.94**	−0.07	0.14	0.12
Eigenvalue	6.46	3.55	1.82	1.35	6.06	3.83	2.63	1.56
Explained variance (%)	37.97	20.85	10.7	7.93	27.53	17.4	15.95	11.82
Cumulative % of variance	37.97	58.82	69.52	77.45	27.53	44.93	60.87	71.69

Note: significant loadings are in bold, TH means Total Hardness.

**Table 4:** Results of *δ*^18^O/*δ*^2^H for groundwater and river water during the wet and dry seasons.

Sample NO.	Dry Season		Wet Season		Sample NO.	Dry Season		Wet Season	
	*δ*^2^H	*δ*^18^O	*δ*^2^H	*δ*^18O^		*δ*^2^H	*δ*^18^O	*δ*^2^H	*δ*^18O^
GW1	−83.4	−9.9	−71.9	−8.3	GW19	−84.2	−10.3	−74.9	−8.8
GW2	−84.7	−10.6	−72.9	−9	GW20	−85.3	−10.7	−76.7	−9.2
GW3	−83.3	−9.9	−72.1	−8.6	GW21	−84.2	−10.3	−76.6	−9.5
GW4	−84.1	−10.1	−73.4	−8.5	GW22	−84	−10.7	−75.6	−8.8
GW5	−84.9	−10.3	−72	−8.6	GW23	−81.2	−9.9	−72.9	−8.3
GW6	−82.6	−9.8	−72.9	−8.7	GW24	−84.2	−10.4	−74.4	−8.9
GW7	−84	−10	−73.6	−8.9	GW25	−83.6	−10.7	−76.3	−9
GW8	−82.9	−10	−74	−8.7	GW26	−83.3	−10.2	−76.9	−9.3
GW9	−83.6	−10.1	−74	−8.9	GW27	−78.4	−9.2	−77.3	−8.6
GW10	−83.8	−10.5	−76	−9	GW28	−82.6	−10	−78.3	−9.4
GW11	−82.5	−9.9	−75.6	−9.4	GW29	−82.1	−10.4	−77.1	−9.2
GW12	−83.3	−10.1	−75.9	−9.2	GW30	−83.5	−10.5	−76.6	−8.8
GW13	−83.8	−10.3	−72.8	−8.7	RW1	−85.9	−10.5	−79.3	−7.8
GW14	−85.1	−10.6	−73.7	−8.5	RW2	−83.9	−9.6	−76.8	−8.8
GW15	−84	−10.3	−74.2	−8.9	RW3	−85.8	−10.1	−76	−8.2
GW16	−82.2	−9.6	−74.6	−8.9	RW4	−84.5	−9.9	−80.1	−8.8
GW17	−84.1	−10	−74.5	−8.7	RW5	−86.4	−10.6	−76.2	−8.4
GW18	−81.3	−9.4	−77.3	−9.1	RW6	−86.6	−10.1	−75.4	−8.4

GW: groundwater, RW: river water.
